# Chronic inflammation is a feature of Achilles tendinopathy and rupture

**DOI:** 10.1136/bjsports-2017-098161

**Published:** 2017-11-08

**Authors:** Stephanie Georgina Dakin, Julia Newton, Fernando O Martinez, Robert Hedley, Stephen Gwilym, Natasha Jones, Hamish A B Reid, Simon Wood, Graham Wells, Louise Appleton, Kim Wheway, Bridget Watkins, Andrew Jonathan Carr

**Affiliations:** 1 NDORMS, Botnar Research Centre, University of Oxford, Nuffield Orthopaedic Centre, Oxford, UK; 2 Faculty of Health and Medical Sciences, University of Surrey, Guildford, UK

**Keywords:** tendon, tendinopathy, Achilles tendon, immunology, orthopaedics

## Abstract

**Background:**

Recent investigation of human tissue and cells from positional tendons such as the rotator cuff has clarified the importance of inflammation in the development and progression of tendon disease. These mechanisms remain poorly understood in disease of energy-storing tendons such as the Achilles. Using tissue biopsies from patients, we investigated if inflammation is a feature of Achilles tendinopathy and rupture.

**Methods:**

We studied Achilles tendon biopsies from symptomatic patients with either mid-portion tendinopathy or rupture for evidence of abnormal inflammatory signatures. Tendon-derived stromal cells from healthy hamstring and diseased Achilles were cultured to determine the effects of cytokine treatment on expression of inflammatory markers.

**Results:**

Tendinopathic and ruptured Achilles highly expressed CD14+ and CD68+ cells and showed a complex inflammation signature, involving NF-κB, interferon and STAT-6 activation pathways. Interferon markers IRF1 and IRF5 were highly expressed in tendinopathic samples. Achilles ruptures showed increased *PTGS2* and *interleukin-8* expression. Tendinopathic and ruptured Achilles tissues expressed stromal fibroblast activation markers podoplanin and CD106. Tendon cells isolated from diseased Achilles showed increased expression of pro-inflammatory and stromal fibroblast activation markers after cytokine stimulation compared with healthy hamstring tendon cells.

**Conclusions:**

Tissue and cells derived from tendinopathic and ruptured Achilles tendons show evidence of chronic (non-resolving) inflammation. The energy-storing Achilles shares common cellular and molecular inflammatory mechanisms with functionally distinct rotator cuff positional tendons. Differences seen in the profile of ruptured Achilles are likely to be attributable to a superimposed phase of acute inflammation and neo-vascularisation. Strategies that target chronic inflammation are of potential therapeutic benefit for patients with Achilles tendon disease.

## Introduction

Achilles tendon disorders including tendinopathy and rupture frequently cause pain and disability in athletes and non-athletic individuals.^1^ These injuries are difficult to treat, require prolonged rehabilitation and have a high frequency of recurrence.^2 3^ The tendon mid-portion is the most frequently injured site^3^; entheseal disease at the calcaneal insertion is less common. Multiple factors contribute to the pathogenesis of tendon disease including the effects of exercise, overuse, genetic predisposition and ageing.^4–7^ While inflammatory mediators are known to contribute to the initiation and progression of tendon disease in the shoulder,^8–11^ the relative importance and role of inflammation is highly debated in energy-storing tendons such as the Achilles, where historically disease was frequently described as ‘degenerative’.^12^

More recent studies have identified immune-competent cells including macrophages, T cells, natural killer and mast cells in human biopsy specimens from non-ruptured chronic tendinopathic Achilles.^13^ However, the phenotypes of these cells are yet to be fully characterised. Apoptosis pathways have also been described as an important signalling cascade implicated in the biology of Achilles tendinopathy.^6^ These studies support the contribution of inflammation in the pathogenesis of Achilles tendon disease, although the precise mechanisms remain to be elucidated. Of importance, the resident stromal fibroblast population that constitutes the major cell type in tendons remains under investigated.

Improved understanding of the cellular and molecular processes orchestrating inflammation in Achilles tendon disease is essential to identify therapeutic targets that address the underlying disease biology. The mechanisms governing the development of chronic inflammation that fails to resolve in persistently symptomatic patients is of particular interest. In the current study, we investigated the cellular and molecular features of inflammation in patient biopsy samples of tendinopathic and ruptured Achilles. We sought to identify if disease of energy-storing tendons such as the Achilles shared common inflammatory mechanisms to those previously identified in positional tendons such as the rotator cuff. We hypothesised that these functionally distinct tendons would share common inflammatory mechanisms.

## Results

### Clinical parameters for the study group

Biopsies from patients with Achilles tendinopathy were collected from 7 female and 10 male patients aged 41–74 years (mean, 50.4±8.8 years) that presented for high-volume injection (HVI). VISA-A scores for patients with Achilles tendinopathy ranged from 25 to 77 (mean, 39.6±15.4). Mean body mass index (BMI) from the Achilles tendinopathy patient group was 29.9 (±6.1).

Tissue biopsy samples were collected from 4 female and 15 male patients that presented to a trauma unit for surgical debridement of an Achilles rupture (n=19). Patients with Achilles rupture were aged 20–67 years (mean, 44.6±11.8 years). VISA-A scores were not available for patients with Achilles ruptures. BMI for the Achilles rupture patient group was (28.8±4).

Healthy hamstring (semitendinosis) tendons were collected from 5 female and 10 male patients aged 18–48 years (mean, 25.5±11 years) during surgical anterior cruciate ligament reconstruction. BMI from this healthy patient group was 24.9 (±2.1).

### Tendinopathic and ruptured Achilles show a complex tissue inflammation signature and increased vascularity

**Figure 1:**
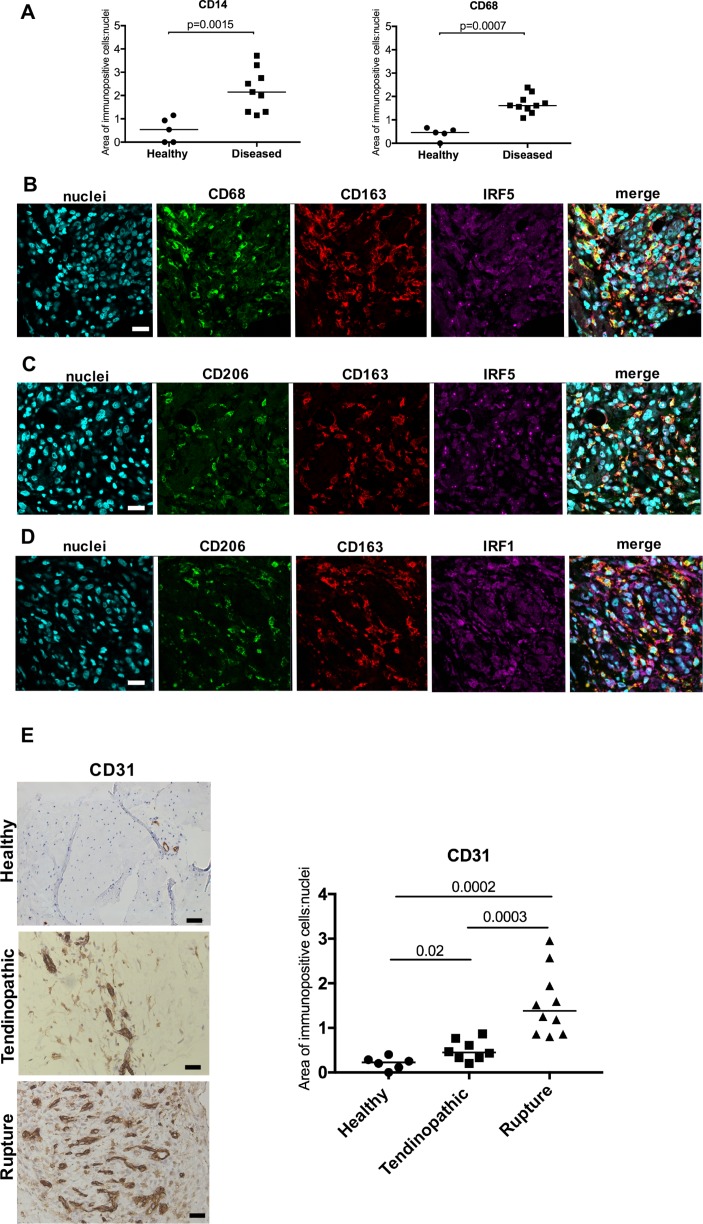
Immunohistochemistry showing the phenotypes of myeloid cells and increased vascularity of diseased Achilles tendons. (A) Quantitative analysis of CD14+ and CD68+ myeloid cells in healthy hamstring and diseased (tendinopathic and ruptured) Achilles tendons. Bars represent median values. (B–D) Representative immunofluorescence images of diseased Achilles tendon sections stained for inflammation activation markers including the interferon pathway (IRF1 and IRF5, purple), the glucocorticoid receptor activation pathway (CD163, red) and STAT-6 pathway (CD206, green). CD68 (green) is a marker of tissue resident macrophages. Cyan shows POPO-1 nuclear stain. Scale bar, 20 µm. (E) Representative images of 3,3′-diaminobenzidine immunostaining (brown) for vascular marker CD31 in healthy hamstring, tendinopathic and ruptured Achilles tendons. Nuclear stain is haematoxylin. Scale bar, 50 µm. Graph shows quantitative analysis of immunostaining for CD31 in healthy hamstring and diseased Achilles tendons.

Using immunohistochemistry, we identified increased expression of CD14+ and CD68+ cells in tendinopathic and ruptured Achilles (collectively grouped as diseased) compared with healthy hamstring tendons (P=0.0015 and 0.0007, respectively) (figure 1A). There was no significant difference in the numbers of CD14+ and CD68+ cells between tendinopathic and ruptured Achilles tendons. We further characterised the activation status of these immune cells in samples of tendinopathic and ruptured Achilles in situ. We used a previously validated panel of antibodies associated with macrophage activation, including proteins implicated in pro-inflammatory pathways (IRF5 and IRF1) and alternative macrophage activation (CD206 and CD163).^8^ Diseased Achilles tendons showed a complex macrophage activation protein signature and expressed markers of interferon (IRF5, IRF1), STAT-6 (CD206) and glucocorticoid receptor (GCR) (CD163) macrophage activation pathways (figure 1B–D). In addition to characterising myeloid cells, we also investigated CD31 as a marker of vascularisation in samples of healthy hamstring, tendinopathic and ruptured Achilles (figure 1E). Quantitative analysis of immunopositive staining showed increased CD31 expression in tendinopathic (P=0.02) and ruptured Achilles (P=0.0002) relative to healthy hamstring tendons. Ruptures showed increased CD31 expression relative to samples of tendinopathic Achilles (P=0.0003) (figure 1E). Isotype control staining of corresponding tendon tissues is shown in online supplementary figure S1.

10.1136/bjsports-2017-098161.supp1 Supplementary file 1

**Figure 2:**
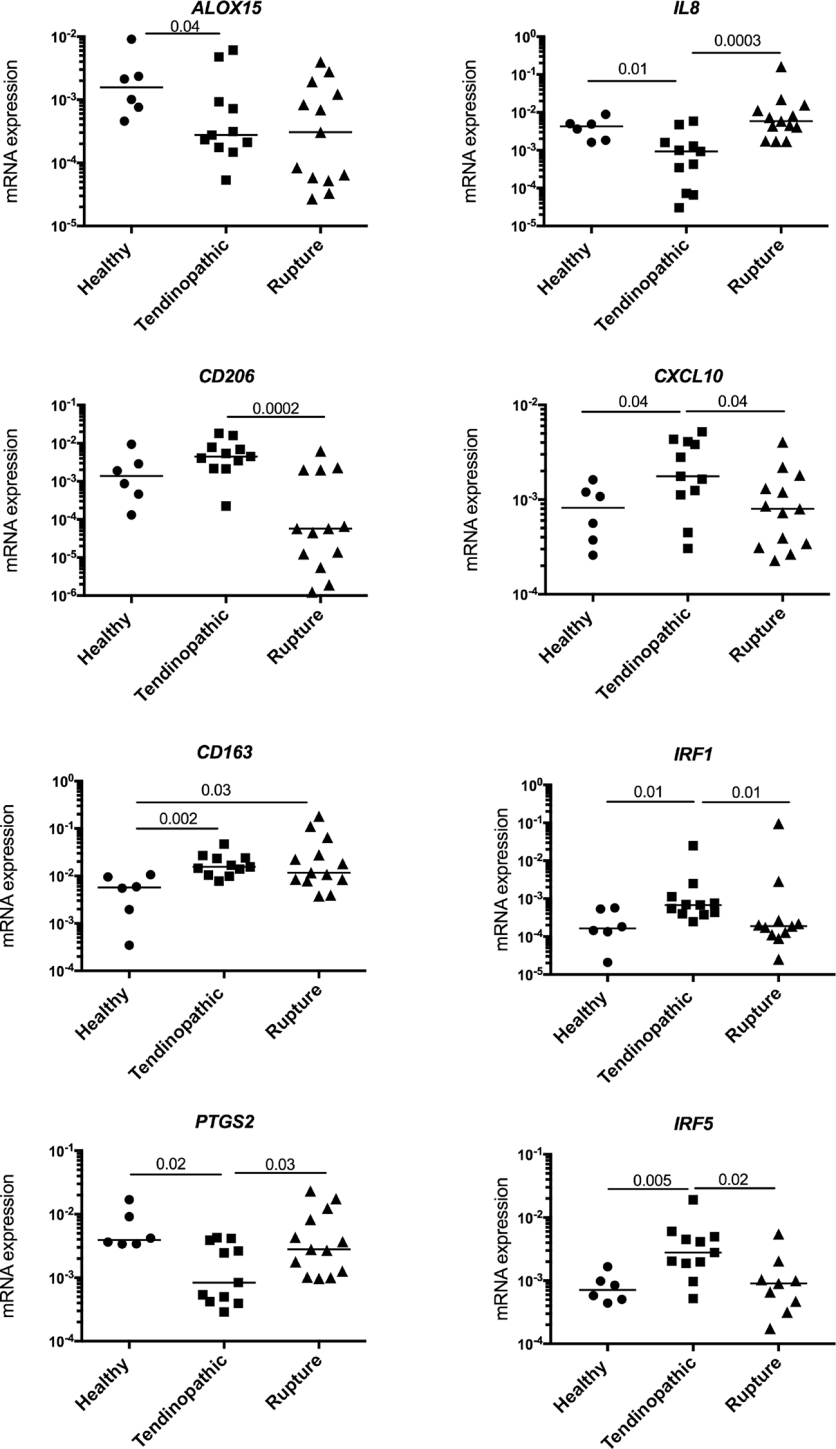
Expression of inflammatory genes in diseased Achilles tendons. Tendinopathic (n=11 donors) and ruptured Achilles (n=13 donors) tissue samples showed a complex inflammation gene signature encompassing activation of NF-κB (*IL-8*, *PTGS2*), interferon (*IRF1*, *IRF5* and *CXCL10*), STAT-6 (*CD206*) and glucocorticoid receptor activation pathways (*CD163*). The inflammation signature of diseased Achilles tendons was compared with healthy hamstring tendons (n=6 donors). Gene expression is shown normalised to β-actin; bars represent median values. mRNA, messenger RNA.

Having identified inflammatory proteins in tendinopathic and ruptured Achilles, we studied gene expression in these samples using a panel of markers implicated in macrophage activation.^8^ In support of the protein signatures identified in these samples, diseased Achilles showed a complex inflammation gene signature with some differences between tendinopathic and ruptured samples (figure 2). *CD163* messenger RNA (mRNA) was highly expressed by tendinopathic and ruptured Achilles (P=0.002 and 0.03, respectively) compared with healthy hamstring tendons. *CD206* mRNA was increased in tendinopathic compared with ruptured Achilles (P=0.0002). Tendinopathic Achilles highly expressed interferon target genes including *IRF1*, *IRF5* and *CXCL10* compared with ruptured and healthy tendons. *ALOX15* implicated in resolving inflammation was decreased in tendinopathic compared with healthy tendons (P=0.04). Ruptures showed increased *IL-8* and *PTGS2* compared with tendinopathic Achilles (P=0.0003 and 0.03, respectively).

### Diseased Achilles tendon tissues express markers of stromal fibroblast activation

**Figure 3:**
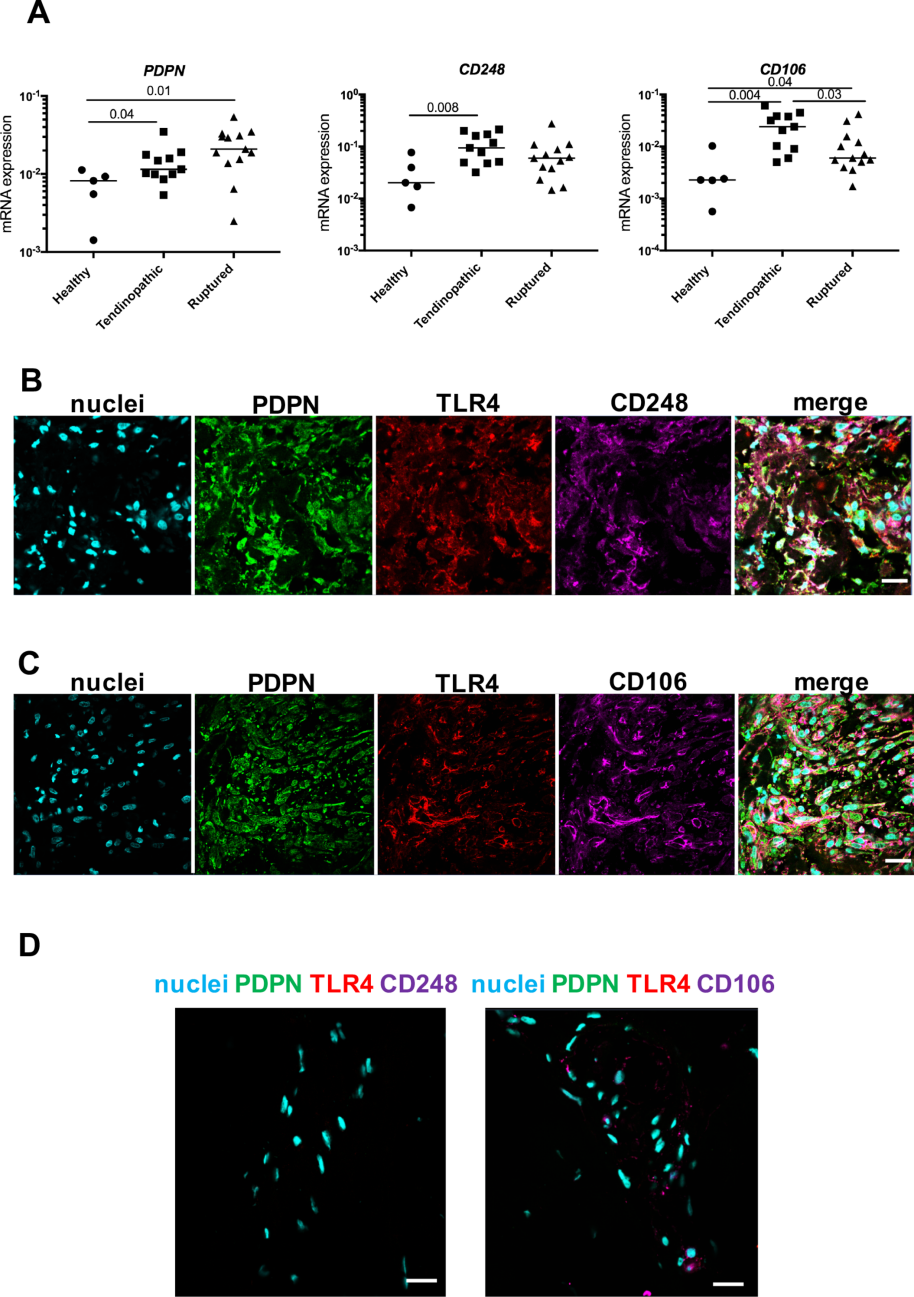
Expression of stromal fibroblast activation markers in healthy hamstring and diseased Achilles tendons. (A) PDPN, CD248 and CD106 mRNA expression in healthy hamstring tendons (n=5 donors), tendinopathic (n=11 donors) and ruptured Achilles tendons (n=13 donors). Gene expression is shown normalised to β-actin; bars represent median values. (B–D) Representative immunofluorescence images of diseased Achilles tendons (tendinopathic) (B) and rupture (C) and healthy hamstring tendon sections (D) stained for markers of stromal activation (PDPN, green; CD248 and CD106, purple) and TLR4 (red). Cyan shows POPO-1 nuclear stain. Scale bar, 20 µm. mRNA, messenger RNA; PDPN, podoplanin; TLR4, toll-like receptor 4.

Having focused on characterising the phenotypes of myeloid cells populating diseased Achilles, we investigated if resident tendon cells in these samples also possessed a pro-inflammatory phenotype. Stromal fibroblast activation (SFA) markers including podoplanin (PDPN), CD106 and CD248 have not been identified in diseased Achilles tendons. We found that *PDPN* mRNA was increased in tendinopathic (P=0.04) and ruptured Achilles (P=0.01) compared with healthy hamstring tendon tissues (figure 3A). *CD106* mRNA was also increased in tendinopathic (P=0.004) and ruptured Achilles (P=0.04) compared with healthy hamstring. *CD248* mRNA was increased in tendinopathic Achilles compared with healthy hamstring (P=0.008). Immunostaining supported increased expression of SFA markers and pro-inflammatory marker toll-like receptor 4 (TLR4) in sections of tendinopathic and ruptured Achilles compared with healthy hamstring tendons (figure 3B–D). In diseased Achilles tendons, PDPN, CD106 and CD248 colocalised with TLR4.

### Diseased Achilles-tendon-derived stromal cells are primed for inflammation

**Figure 4:**
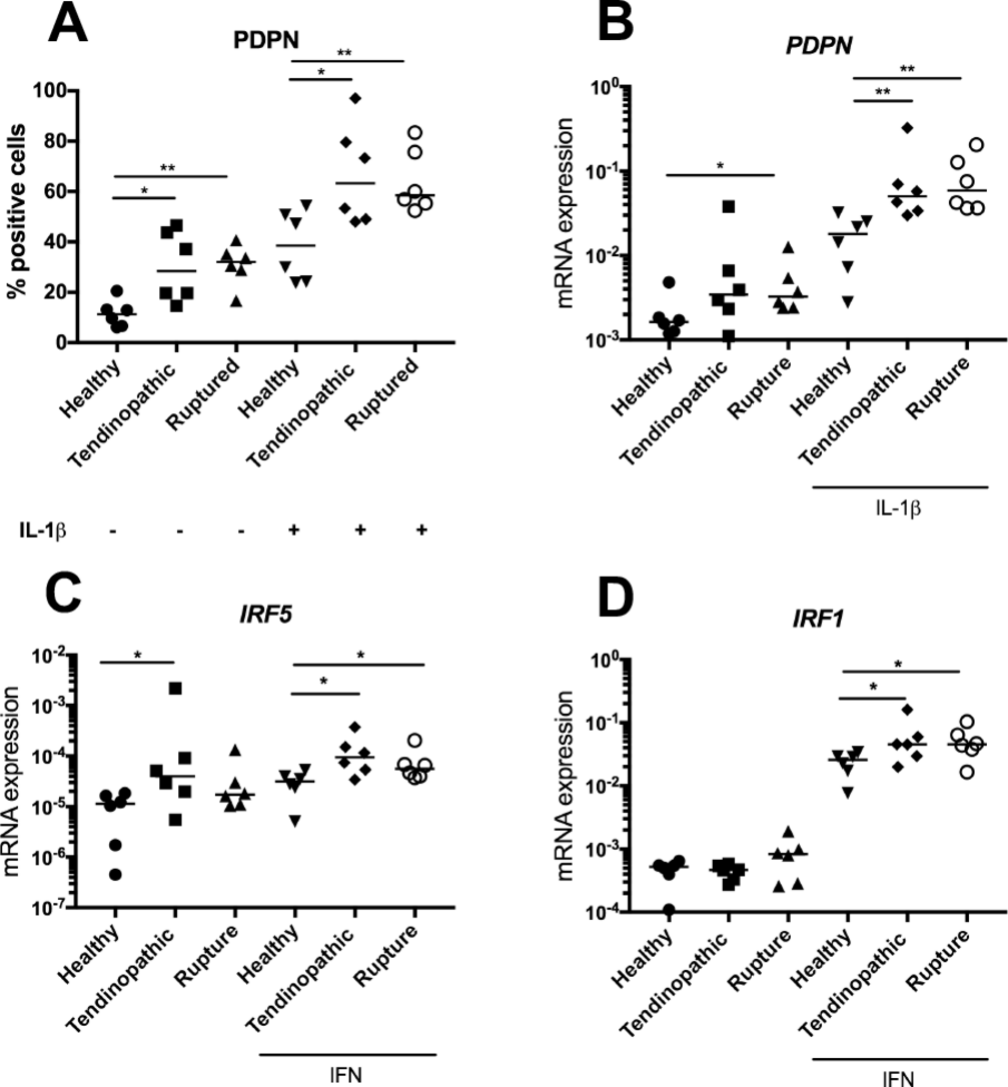
Expression of markers of inflammation in cultured tendon-derived stromal cells after cytokine treatment. Tendon stromal cells were derived from healthy hamstring (n=6 donors), tendinopathic (n=6 donors) or ruptured Achilles (n=6 donors). (A) PDPN expression after IL-1β treatment (10 ng/mL) for 24 hours determined by flow cytometry. (B) Expression of *PDPN* mRNA after IL-1β treatment (10 ng/mL) for 24 hours. Expression of interferon target genes *IRF5* (C) and *IRF1* (D) after IFNγ treatment (20 ng/mL) for 24 hours. Gene expression is shown normalised to β-actin; bars represent median values. **P<0.01, *P<0.05. mRNA, messenger RNA; PDPN, podoplanin.

Markers of inflammatory pathways and fibroblast activation detected in tendinopathic/ruptured Achilles tissues were subsequently studied *in vitro* to compare the effects of cytokine treatment on tendon-derived stromal cells isolated from healthy hamstring and diseased tendinopathic and ruptured Achilles tendons. PDPN protein was increased in cells isolated from tendinopathic and ruptured Achilles relative to healthy hamstring under unstimulated conditions (P=0.01 and P=0.004, respectively) (figure 4A). Stimulation with IL-1β for 24 hours further induced PDPN in healthy and diseased tendon cells. IL-1β-stimulated tendinopathic and ruptured Achilles showed profound induction of PDPN relative to respective healthy tendon cells (P=0.04 and 0.004, respectively) (figure 4A). Similar observations were made when *PDPN* mRNA expression was determined after IL-1β stimulation of healthy and diseased tendon cells (figure 4B). *IRF5* mRNA was increased in cells isolated from tendinopathic Achilles relative to healthy hamstring under unstimulated conditions (P=0.026) (figure 4C). IFNγ treatment induced expression of interferon target genes in tendon cells isolated from healthy hamstring and diseased Achilles. IFNγ treatment markedly induced *IRF5* mRNA in cells isolated from tendinopathic and ruptured Achilles compared with healthy hamstring (P=0.016 and 0.026, respectively) (figure 4C). The same treatment also induced *IRF1* mRNA in cells isolated from tendinopathic and ruptured Achilles compared with healthy hamstring (P=0.04) (figure 4D).

## Discussion

The role of inflammation in the aetiopathogenesis of tendon disease is a subject of ongoing debate. During the past 20 years, tendon disease has been characterised as a ‘degenerative’ process devoid of inflammation. Recently, this paradigm has been challenged, suggesting that it may disregard a complex role for inflammation in tendon disease. Coupled with improved scientific methodologies and collection of well-phenotyped patient biopsy material, our understanding of tendon inflammatory processes has advanced in recent years. Recent studies have improved understanding of the phenotypes of immune cells identified in biopsy samples of diseased human tendons.^8^ Other work has also identified that resident stromal fibroblasts from diseased human tendons show a pro-inflammatory phenotype,^11^ illustrating that non-immune cells are also implicated in tendon inflammatory processes. Understanding the phenotypes of immune cells and tendon stromal populations is critical to advancing knowledge of inflammation in the pathobiology of Achilles tendon disease.

The present study investigated the cellular and molecular features of inflammation in diseased Achilles tendons. Using tissues derived from well-phenotyped patient cohorts, we identified that inflammation is a feature of both mid-portion Achilles tendinopathy and rupture. The pathways determining activation of macrophages to be classified as M1 or M2 phenotypes have recently been revised to reflect the key signalling pathways and receptors in distinct activation pathways. These include pro-inflammatory interferon and NF-κB pathways, the pro-fibrotic STAT-6 pathway and GCR activation pathway.^14^ We investigated expression of target molecules from each of these pathways in healthy hamstring and diseased Achilles tendon tissues. Tissue samples from Achilles patient cohorts showed a complex inflammation signature, expressing target molecules from interferon, NF-κB, STAT-6 and GCR activation pathways. Both tendinopathic and ruptured Achilles tendons expressed CD206 and CD163, suggestive of established (chronic) inflammation and tissue repair. We identified some differences in the inflammatory profiles depending on the type of disease. Tendinopathic Achilles showed increased expression of interferon target genes and proteins including IRF1, IRF5 and CXCL10. We previously identified that an interferon signature similar to that found in tendinopathic Achilles tendons was a feature of tissue biopsies from tendinopathic rotator cuff.^8^ In contrast, ruptured Achilles tendons highly expressed NF-κB target genes *PTGS2* and *IL-8* and showed increased vascularity. Genes associated with angiogenesis including vascular endothelial growth factor have been identified in samples of diseased Achilles tendons.^15^ Furthermore, IL-8 is a significant promoter of angiogenesis.^16^ This NFκB signature of Achilles tendon ruptures was not a feature of tissue samples collected from patients with large-massive rotator cuff tendon tears.^8^ Table 1 summarises the comparative features of disease in functionally distinct tendons such as the Achilles and rotator cuff. While patients with Achilles rupture may have had pre-existing tendon disease prior to rupture, the timing of tissue collection relative to an acute event such as tendon rupture is an important factor in the interpretation of these findings. The differences in inflammation signature between torn Achilles and rotator cuff tendons are likely attributable to the consequences of recent trauma and earlier clinical presentation of patients with Achilles rupture. Conversely, symptom duration and subsequent surgical repair is frequently more protracted (months to years) in patients with rotator cuff tendon tears (table 1). This phase of acute inflammation and neo-angiogenesis may partly explain the higher success rate of repair of Achilles tendon ruptures compared with repair of torn rotator cuff tendons.^17^

**Table 1:** Comparative features of disease in functionally distinct tendons

Feature	Achilles tendon disease	Shoulder tendon disease
Symptom duration	Acute or chronic	Frequently chronic
Type of disease	Tendinopathic or ruptured	Tendinopathic or torn
Typical time to clinical presentation	Days to weeks (rupture), months to years (tendinopathic)	Months to years
Inflammation changes with type of disease	Yes	Yes
Inflammation changes after treatment in asymptomatic patients	Unknown	Yes

**Figure 5:**
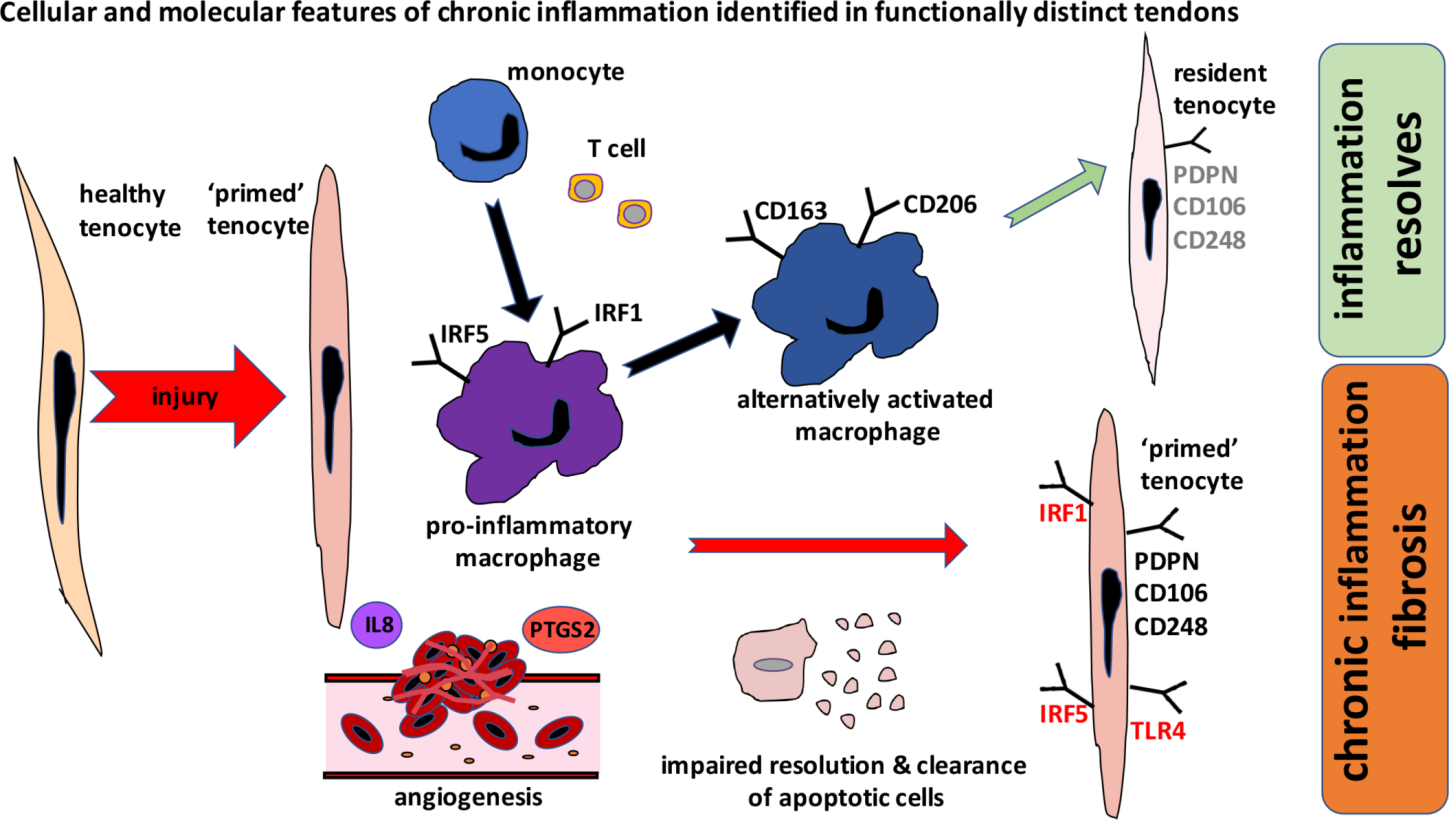
Schematic summarising the cellular and molecular features of chronic inflammation identified from cross-sectional assessments of functionally distinct tendons. Macrophages in diseased tendons show a mixed signature and express pro-inflammatory macrophage markers (IRF1, IRF5) and markers of alternative macrophage activation including CD206 and CD163. After exposure to inflammatory stimuli, diseased tendon cells become ‘primed’ and express markers of stromal fibroblast activation including PDPN and adopt a more rounded morphology, reflecting a phenotypic shift in their inflammatory profile. Other pro-inflammatory molecules expressed by tendon cells include damage-associated molecular pattern TLR4, IRF1 and IRF5. Achilles tendon ruptures show increased vascularity and highly express *interleukin-8* and PTGS2. Chronic inflammation and fibrosis develop due to impaired resolution of inflammation and failure of clearance of apoptotic cells. With successful resolution, expression of pro-inflammatory mediators is moderated, although some degree of stromal fibroblast activation persists. This stromal ‘memory’ may sensitise tendon cells and increase susceptibility to further episodes of inflammation and recurrent tendon disease. PDPN, podoplanin; TLR4, toll-like receptor 4.

Having characterised the phenotypes of myeloid cells in diseased Achilles tissues, we next investigated the phenotype of resident tendon stromal fibroblasts, (tenocytes) in tissues and cells derived from these patient cohorts. We previously identified that tissues and cells isolated from diseased shoulder tendons highly expressed SFA markers, suggestive of a sustained change in their phenotype as a consequence of exposure to inflammation.^11^ To our knowledge, the phenotype of fibroblasts populating diseased Achilles tendons has not been described. We discovered that tissue samples from patients with Achilles tendinopathy and rupture also highly expressed PDPN, CD106 and CD248. A recent study investigating inflammation in cultured stromal fibroblasts isolated from torn rotator cuff tendons identified that diseased tendon cells were ‘primed’ for inflammation.^8^ In the present study, we investigated if cells isolated from patients with disease of functionally distinct Achilles tendons were similarly ‘primed’. IL-1β or IFNγ stimulation of healthy and diseased tendon stromal cells induced expression of respective target genes compared with untreated control cells. Notably, cytokine-treated tendon stromal cells from diseased Achilles showed profound induction of PDPN and inflammatory proteins IRF1 and IRF5 compared with healthy hamstring tendon stromal cells. Collectively, our data support the concept that tissues and cells from diseased Achilles tendons undergo phenotypic change and may become ‘primed’ after exposure to inflammation. The cellular and molecular features of chronic inflammation common to functionally distinct tendons are summarised in figure 5, whereby impaired resolution of inflammation and failure to clear apoptotic cells sustains chronic inflammation and fibrosis. Conversely, with successful resolution, expression of pro-inflammatory mediators is moderated, although some degree of SFA persists.^8 11^ This stromal ‘memory’ may sensitise tendon stromal cells and increase susceptibility to further episodes of inflammation and recurrent tendon disease.

There are potential limitations of using hamstring tendon as a comparator to diseased Achilles, including differences in donor age and tendon type. However, hamstring tendons were collected from healthy donors without history of tendinopathy. This is preferable to using cadaveric Achilles tendon tissues, where the tissues can be affected by postmortem change and little is known about the health status of the tissue. In addition, as sex distribution was not evenly matched between Achilles tissue cohorts, it was necessary to pool data for men and women.

The findings from our study demonstrate that tissues from patients with tendinopathic and ruptured Achilles tendons both show evidence of chronic inflammation. Moreover, functionally distinct tendons such as the Achilles and rotator cuff share common cellular and molecular features. We identify slight differences in pro-inflammatory profiles between tendinopathic and ruptured Achilles tendons and suggest that this difference is attributable to superimposed acute inflammation and increased vascularisation, occurring after recent Achilles tendon rupture. We propose that treatment strategies that target non-resolving inflammation are of potential therapeutic benefit for patients with Achilles tendon disease.

## Materials and methods

### Study design

The study objective was to investigate the cellular and molecular features of inflammation in Achilles tendon disease. Tissue samples were collected from patient cohorts with mid-portion Achilles tendinopathy or rupture. We investigated tissue inflammation signatures in these patient cohorts compared with healthy hamstring tendons. Inflammatory pathways were subsequently investigated in cultured stromal fibroblasts derived from healthy and diseased tendons. Sample size justification was derived from previous studies that were sufficiently powered to study inflammation in diseased tendon tissues and cells.^8 11^ For histology and immunostaining, a single-blinded investigator acquired images. Sample sizes and experimental replicates are outlined in figure legends.

### Collection of tendon tissues

Patients with Achilles tendinopathy were recruited from a sports medicine clinic (n=17). Patients presenting to the sports clinic had been symptomatic for months and failed a standard 3-month eccentric training programme for Achilles tendinopathy. Ultrasonography was performed to confirm the presence of Achilles tendinopathy and suitability for HVI for patients who have failed conservative management, as this procedure represents the next step in standard treatment. Patients completed the VISA-A scoring system,^18^ a validated clinical outcome measure scoring from 0 (severe disease) to 100 (normal function). Achilles tendon biopsies were collected from patients that presented for HVI. This procedure involves injecting 10 mL 0.5% bupivicaine and 30 mL saline into the pre-Achilles space. Achilles tendon biopsies were obtained via percutaneous ultrasound-guided biopsy under local anaesthesia prior to HVI at the site of ultrasonographic abnormality. The specimen was collected using a 14G trucut biopsy needle inserted into the diseased mid-portion of the Achilles. This validated biopsy technique is adapted from a previously described protocol.^19^

Patients with Achilles tendon rupture were recruited from a trauma unit (n=19). Tissue biopsy samples of Achilles tendon ruptures were collected between 36 and 48 hours after tendon rupture. Full informed consent according to the Declaration of Helsinki was obtained from all patients participating in the study. Exclusion criteria included previous intratendinous corticosteroid/platelet-rich plasma/stem cell injection, extracorporeal shockwave therapy or systemic steroid or methotrexate treatments. Diabetic patients and those receiving systemic anticoagulant therapy were also excluded from the study.

Healthy hamstring (semitendinosis) tendons were collected from 15 patients undergoing surgical anterior cruciate ligament reconstruction.

### Tendon tissue sample processing

#### Immunohistochemistry and immunofluorescence

Tendon tissues were fixed in 10% formalin, processed using a Leica ASP300S tissue processor and embedded in paraffin wax. Tissues were sectioned at 6 µm using a rotary RM2135 microtome (Leica Microsystems) onto glass slides.

#### Gene expression

Healthy hamstring and diseased Achilles tendon tissue samples were snap-frozen in liquid N_2_ and stored at −80°C until RNA extraction.

### Immunostaining for markers of macrophages and SFA

Antigen retrieval and single-staining immunohistochemistry for CD14 and CD68 were performed using previously published protocols.^8^ For multiplex immunofluorescence staining and image acquisition, protocols were adapted from Dakin *et al* 8 using primary antibodies listed in online supplementary table 1. Isotype controls were mouse IgG_1_, IgG_2a_, IgG_2b_, IgG_3_ and IgM and rabbit immunoglobulin (Dako) ready-to-use antibodies (online supplementary figure S1). Images from stained sections were captured using a Zeiss LSM 710 confocal microscope using previously a published protocol.^8^

### Cytokine treatment of tendon-derived stromal cells

IL-1β and IFNγ respectively induce NF-κB and IFN target genes expressed in diseased shoulder tendon tissues^8 11^ and were also identified in diseased Achilles tendon tissues in the current study. We therefore investigated if treatment of cultured tendon cells with these cytokines induced more profound expression of SFA markers and NF-κB and IFN target genes in diseased relative to healthy tendon-derived cells. Tendon-derived cells were isolated from healthy hamstring and diseased Achilles tendons using previously described protocols^11^; passage 1–3 cells were used for all experiments. Cells were grown until 80% confluence prior to stimulation with IL-1β (10 ng/mL, Sigma) or IFN gamma (20 ng/mL, BioLegend) in medium (DMEM F12, Lonza) containing 1% heat-inactivated human serum (Sigma). Non-treated (vehicle only) cells served as experimental controls. After cytokine/vehicle treatment, cells were incubated at 37°C and 5% CO_2_ for 24 hours until experimental harvest for mRNA or flow cytometry.

### RNA extraction from tendons

Protocols for RNA extraction, complementary DNA synthesis and quantitative PCR are described elsewhere.^8^ Validated human primers are listed in online supplementary table 2. Reactions were run in duplicate for each gene on a ViiA7 qPCR machine (Applied Biosystems). Results were calculated using the DDCt method using reference genes for human β-actin and glyceraldehyde 3-phosphate dehydrogenase. Results were consistent using these reference genes, and data shown are normalised to β-actin.

### Flow cytometry of tendon-derived stromal cells

Flow cytometry was performed as previously described using a BD LSR Fortessa instrument.^11^ Antibody and isotype cocktails were prepared as listed in online supplementary table 3. Data analysis was performed using FlowJo software (Treestar); tendon cell populations were gated on CD45^−^ and CD34^−^ cells.

### Statistical analysis

GraphPad Prism 6 (GraphPad Software) was used to perform statistical analysis. Normality was tested using the Shapiro-Wilk normality test. Pairwise Mann-Whitney U tests were used to test for differences in expression of CD14 and CD68 in healthy and diseased tendons. Kruskal-Wallis tests followed by pairwise post hoc Mann-Whitney U tests were used to compare mRNA expression of macrophage and SFA genes in healthy hamstring and in diseased tendinopathic and ruptured Achilles tendons. Pairwise Mann-Whitney U tests were used to determine differences between mRNA expression of macrophage and SFA target genes in cytokine-treated healthy and diseased tendon cells. Statistical significance was set at P<0.05.

**What are the findings?**

- Chronic inflammation is a feature of both mid-portion Achilles tendinopathy and rupture.
- Pro-inflammatory profiles differ slightly in ruptured tendons; this is likely to be due to acute inflammation and increased vascularisation resulting from recent trauma.
- Although the Achilles and rotator cuff tendons are functionally distinct, they share common cellular and molecular inflammatory disease mechanisms.

**How might it impact on clinical practice in the future?**

- Therapeutic strategies must address the underlying biology of Achilles tendon disease if treatment is to be successful.
- Strategies that target inflammation are of potential therapeutic benefit for patients with chronic Achilles tendon disease.
- New therapeutic approaches are required to promote resolution of inflammation in chronic tendinopathy.
