# Supplementary material for: Chronic inflammation is a feature of Achilles tendinopathy and rupture
Source: Br J Sports Med. 2017 Nov 8;52(6):359–67. doi: 10.1136/bjsports-2017-098161 (PMC5867427; doi:10.1136/bjsports-2017-098161)
Supplement: Supplementary file 1 [file bjsports-2017-098161supp001.pdf]

SUPPLEMENTARY INFORMATION

Table S1. Primary antibodies used for immunohistochemistry and immunofluorescence

Antibody	Clone	Isotype	Species	Dilution
IRF5 Proteintech 10547-1-AP		IgG	rabbit	1:100
CD163 LS-Biosciences LS_C174770	34B	IgG_{2a}	mouse	1:150
CD206 Abcam Ab117644	5C11	IgG_1	mouse	1:200
CD68 Dako M0814	KP1	IgG_1	mouse	1:50
CD14 Proteintech 17000-1-AP		IgG	rabbit	1:50
Podoplanin (PDPN) Abcam Ab10288	18H5	IgG_1	mouse	1:100
CD248 (TEM1) Abcam Ab204914	EPR17081	IgG	rabbit	1:1000
CD106 (VCAM-1) LS-Biosciences LS_C313019		IgG	rabbit	1:100
TLR4 Abcam Ab22048	76B357.1	IgG_{2b}	mouse	1:200
CD31 Abcam Ab187377	[C31.3]	IgG_1	mouse	1:150

Table S2. Primers used for quantitative polymerase chain reaction.

Gene Symbol	Gene Description	Sequence
β-actin	Actin, beta	Qiagen QT00095431
GAPDH	glyceraldehyde-3-phosphate dehydrogenase	Qiagen QT00079247
PDPN	Podoplanin	Qiagen QT01015084
CD248	CD248 molecule (endosialin)	Qiagen QT00216356
CD106	CD106 molecule (VCAM)	Qiagen QT00018347
PTGS2	Prostaglandin-endoperoxide synthase 2	Qiagen QT00040586
IL-8 (CXCL8)	Interleukin 8	Qiagen QT00000322
IRF1	Interferon regulatory factor 1	CTGTGCGAGTGTACCGGATG<br>ATCCCCACATGACTTCCTCTT
IRF5	Interferon regulatory factor 5	GGGCTTCAATGGGTCAACG<br>GCCTTCGGTGTATTTCCCTG
CXCL10	Chemokine (C-X-C motif) ligand 10	TCCACGTGTTGAGATCATTGC<br>TCTTGATGGCCTTCGATTCTG
ALOX15	Arachidonate 15-lipoxygenase	GGGCAAGGAGACAGAACTCAA<br>GCCGATCCACACGGAGTACT
CD206	CD206 molecule (mannose receptor)	Qiagen QT00012810
CD163	CD163 molecule	Qiagen QT00074641

Table S3. Antibodies used for flow cytometry. All antibodies were diluted 1:50 for staining.

Antibody	Catalog #	Clone
PDPN Alexa Fluor 488 anti-human	337005 BioLegend	NC08
Alexa Fluor 488 Rat IgG2aκ isotype	400525 BioLegend	RTK2758
CD34 PerCP/Cy5.5 anti-human	343521 BioLegend	581
PerCP/Cy5.5 mouse IgG1κ isotype	400149 BioLegend	MOPC-21
CD45 BV605 anti-human	304041 BioLegend	H130
BV605 mouse IgG1κ isotype	400161 BioLegend	MOPC-21

Figure S1

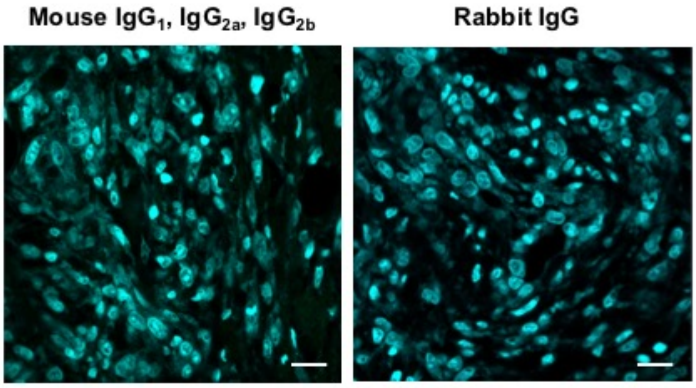

Figure S1 Isotype control staining of diseased Achilles tendons. Representative confocal immunofluorescence images showing merged image of diseased Achilles tendon sections stained with Isotype control antibodies for mouse IgG1, IgG2a, IgG2b and rabbit IgG fractions. Cyan represents POPO-1 nuclear counterstain. Scale bar, 20μm.
